# Uptake/Efflux Transport of Tramadol Enantiomers and *O*-Desmethyl-Tramadol: Focus on *P*-Glycoprotein

**DOI:** 10.1111/j.1742-7843.2009.00428.x

**Published:** 2009-09

**Authors:** Mouna Kanaan, Youssef Daali, Pierre Dayer, Jules Desmeules

**Affiliations:** Department of Anaesthesiology, Clinical Pharmacology and Toxicology and Multidisciplinary Pain Center, Pharmacology and Intensive care, Geneva University Hospitals, Faculty of Medicine, University of GenevaCH-1211 Geneva 14, Switzerland

## Abstract

**Abstract:**

The analgesic effect of tramadol (TMD) results from the monoaminergic effect of its two enantiomers, (+)-TMD and (−)-TMD as well as its opioid metabolite (+)-*O*-desmethyl-tramadol (M1). *P*-glycoprotein (*P*-gp) might be of importance in the analgesic and tolerability profile variability of TMD. Our study investigated the involvement of *P*-gp in the transepithelial transport of (+)-TMD, (−)-TMD and M1, using a Caco-2 cell monolayer model. The bidirectional transport of racemic TMD and M1 (1–100 µM) across the monolayers was investigated at two pH conditions (pH 6.8/7.4 and 7.4/7.4) in the presence and absence of *P*-gp inhibitor cyclosporine A (10 µM) and assessed with the more potent and specific *P*-gp inhibitor GF120918 (4 µM). Analytical quantification was performed by liquid chromatography coupled to the fluorescence detector. A net secretion of (+)-TMD, (−)-TMD and M1 was observed when a pH gradient was applied (TR: *P*_app_(B − A)/*P*_app_(A − B): 1.8–2.7; P < 0.05). However, the bidirectional transport of all compounds was equal in the non-gradient system. In the presence of *P*-gp inhibitors, a slight but significant increase of secretory flux was observed (up to 26%; P < 0.05) at both pH conditions. In conclusion, (+)-TMD, (−)-TMD and M1 are not *P*-gp substrates. However, proton-based efflux pumps may be involved in limiting the gastrointestinal absorption of TMD enantiomers as well as enhancing TMD enantiomers and M1 renal excretion. A possible involvement of uptake carriers in the transepithelial transport of TMD enantiomers and M1 is suggested.

Tramadol hydrochloride (TMD) is a centrally acting analgesic structurally related to codeine and morphine [1]. Experimental and clinical studies have demonstrated the analgesic efficacy and tolerability of TMD in acute and chronic nociceptive and neuropathic pain of both malignant and non-malignant origin [2–4]. TMD is mainly metabolised by *O-* and *N*-demethylation and by conjugation reactions, forming glucuronides and sulfates. The *O-*demethylation of TMD to its opioid active moiety *O*-desmethyl-tramadol (M1) is catalysed by cytochrome P450 (CYP) 2D6, whereas *N-*demethylation to *N-*desmethyl-TMD (M2) is catalysed by CYP2B6 and CYP3A4 (fig. 1) [5,6]. Both enantiomers of TMD, (+)-TMD and (−)-TMD, as well as its major metabolite M1 contribute to its analgesic activity via different mechanisms. M1 acts mainly as a µ opioid agonist (Ki: 0.0034 µmol/l) whereas (+)-TMD and (−)-TMD inhibit serotonin and norepinephrine reuptake, respectively (Ki: 0.53 and 0.43 µmol/l), thus enhancing the inhibitory effect on pain transmission in the spinal cord [7,8]. The complementary and synergistic actions of the three compounds confer a unique pharmacodynamic profile to TMD [9].

**Fig. 1:**
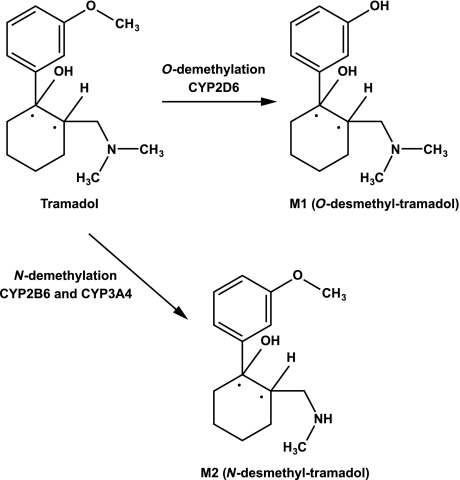
Major *in vivo* metabolic pathways for tramadol.

However, wide variability in the pharmacokinetic/pharmacodynamic properties of TMD has been shown. This has been partly ascribed to CYP2D6 polymorphism, as the µ opioid TMD effect is mainly M1-dependent [10–13]. Stereoselective *O-* and *N*-demethylation, as well as renal elimination of TMD, have also been well-characterized [14,15].

*P*-glycoprotein (*P*-gp/MDR1/ABCB1) is a broad-spectrum efflux transporter that limits substrate xenobiotic exposure in the body, thus playing a crucial role in drug pharmacokinetics and biodisposition [16]. It has been shown to stereo-selectively transport numerous drug enantiomers in several *in vitro* and *in vivo* studies [17–20]. The potential stereo-selective transport of TMD enantiomers by an ABC efflux drug transporter as well as a preferential transport of TMD and M1 have not yet been investigated. These may play non-negligible roles in the biodisposition of each compound and, therefore, the final expected central analgesic effect of the drug racemate and its active opioid metabolite M1. Several opioid analgesics have been shown to be substrates as well as inhibitors of *P*-gp in studies using the *in vitro* Caco-2 monolayer model [21–23].

The present study assessed whether the two enantiomers of TMD and its metabolite M1 were subject to an active efflux transport across biological membranes, focusing mainly on the efflux transporter *P*-gp.

## Materials and Methods

### Materials

Caco-2 cells (TC7 clone) were kindly provided by Martin Rumbo, PhD (ISREC, Swiss Institute for Experimental Cancer Research, Lausanne, Switzerland). 32-(*N*-morpholino)ethane-sulfonic acid (MES) was purchased from AppliChem GmbH (Darmstadt, Germany), penicillin–streptomycin from Sigma Aldrich GmbH (Steinheim, Germany), non-essential amino acids from Biochrom AG (Berlin, Germany) and all other cell culture reagents from Gibco BRL (Scotland, England). [^3^H]-mannitol and Pico-Fluor 15 medium were purchased from Perkin Elmer Life Sciences (Zaventem, Belgium). TMD and *O*-desmethyl-TMD, cyclosporine A and [*N-*(4-[2-(1,2,3,4-tetrahydro-6,7 dimethoxy-2-isoquinolinyl) ethyl]-phenyl)-9,10-dihydro-5-methoxy-9-oxo-4-acridine carboxamide] (GF120918) were kindly provided by Grünenthal GmbH (Stolberg, Germany), Novartis Pharma (Basel, Switzerland) and GlaxoSmithKline (Stevenage, UK), respectively.

### Cell culture

Caco-2 cells (TC7 clone) were used at passages 40–55. Cells were cultured in Dulbecco's modified Eagle's medium (DMEM Glutamax, Gibco BRL) supplemented by 10% foetal bovine serum (FBS, Gibco BRL), 1% non-essential amino acids (NEA, Biochrom AG) and 100 U/ml penicillin and 100 µg/ml streptomycin (Sigma Aldrich) at 37° in a humidified atmosphere with 5% CO_2_. At 85–95% confluency, Caco-2 cells were treated with 0.25% trypsine-EDTA (Gibco BRL) and seeded at a density of 65,000 cells/cm^2^ on polycarbonate membranes of Transwells (12 mm diameter, 1.13 cm^2^, 0.4 µm pore size, 12-well plates; Costar, Cambridge, MA), previously equilibrated for 1 hr. Medium was changed the day after seeding and every other day thereafter (apical volume (A): 0.5 ml, basolateral volume (B): 1.5 ml). Monolayers were used for transport studies 20–21 days post-seeding to allow full maturation of the cells, including *P*-gp expression and appropriate tight junctions.

### Measurement of transepithelial electrical resistance (TEER)

TEER was checked every 5 days during the 21-day monolayers maturation. Prior to bidirectional transport studies, medium was removed from both apical and basolateral chambers and monolayers were rinsed three times with the transport buffer Hank's balanced salt solution (HBSS) supplemented with 25 mM *N*-(2-hydroxylethyl)piperazine-N′-2ethane-sulfonic acid (HEPES) (Gibco BRL) and pH-adjusted to 7.4 with 0.5 M NaOH. Cells were equilibrated in the same buffer for 1 hr and the integrity of each monolayer was checked by measuring its transepithelial electrical resistance (TEER) with a Millicell-ERS ohmmeter (Millipore Corp., Bedfort, MA). Resistance was also checked immediately after the transport experiments.

### Transmission electron microscopy and Western blotting of *P*-gp

Transmission electron microscopy and Western blotting of *P*-gp were performed in our earlier study in the context of the validation of our Caco-2 cell model. Histological examination showed a continuous, differentiated cell monolayer presenting microvillus on the apical cell surface, interdigitations, numerous desmosomes (maculae adherents) and tight junctions. Western blot analysis revealed a C219 antibody-reactive band of 170 kD corresponding to *P*-gp expression [24].

### Transport studies

The bidirectional transport of the two enantiomers of TMD and M1 (1–100 µM) was investigated at pH 6.8/7.4 and 7.4/7.4 in the presence and absence of cyclosporine A, at respectively 1–100 and 4–400 times the plasma concentrations (Cmax) that produce a clearcut analgesic effect after a 100 mg dose of TMD *in vivo*[25]. The pH gradient condition (6.8/7.4) was used to examine whether pH-mediated efflux mechanisms are involved in TMD and M1 transport across pH gradients that exist in intestinal and kidney transport [26,27]. The bidirectional transport of all molecules was assessed at the lowest concentration (1 µM) with GF120918 at physiological pH (7.4/7.4) to confirm *P*-gp interaction with the drugs tested. Indeed, GF120918 does not inhibit the multi-resistance proteins (MRPs) [28]. It may inhibit the breast cancer resistance protein (BCRP) but this transporter is expressed in very low extent in Caco-2 cells [29].

*pH 6.8/7.4 condition:*After measurement of TEERs, HBSS buffer was removed from each chamber. Apical to basolateral (A-B) transport was initiated by replacing basolateral (B) buffer with 1.5 ml of fresh HBSS supplemented with 25 mM HEPES (Gibco BRL) and pH-adjusted to 7.4 with NaOH 0.5 M, and replacing apical (A) buffer with 0.5 ml of the drug solution in HBSS supplemented with 10 mM MES (AppliChem GmbH), pH-adjusted to 6.8 with 0.5 M NaOH. Into another insert, B–A transport was initiated by replacing (A) buffer with 0.5 ml of fresh HBSS/MES pH 6.8 and (B) buffer with the drug solution in HBSS/HEPES (1.5 ml). For the *P*-gp inhibition studies, the inhibitor was present in both chambers. Cyclosporine A and GF120918 were used at 10 and 4 µM, respectively. Samples (150 µl for TMD and 100 µl for M1) were removed from each receiver chamber at various times (30, 60, 90, 120 and 180 min) and replaced with buffer to maintain constant volumes. The 3-hr transport studies were performed at a constant agitation rate (50 rpm) using a circular shaker (type SSM1, Stuart®) in an incubator (37°, 5% CO_2_and humidified atmosphere).

*pH 7.4/7.4 condition:* As described for the pH 6.8/7.4 condition except that (A) and (B) buffers and drug solutions were made with HBSS supplemented with 25 mM HEPES pH 7.4.

After the transport studies, all aliquots were stored at –20° until analysis.

### Paracellular transport of [^3^H]-mannitol

Apical to basolateral permeability (A-B) of [^3^H]-mannitol, a radio-labelled paracellular marker (activity: 0.5 µCi/ml), was measured over 3 hr to monitor the integrity of monolayers tight junctions. Samples taken from the basolateral side (100 µl) at various times were counted in a Pico-Fluor 15 medium using a liquid scintillation counter (Packard Instruments).

### Analytical method

Analysis of (+)-TMD, (−)-TMD and M1 was performed by liquid chromatography coupled to the fluorescence detector. A stereo-selective method was applied for (+)-TMD and (−)-TMD determination using a Chiral AGP column (100 × 4.0 mm), as previously described [30] with slight modifications. The mobile phase consisted of a mixture of acetonitrile and phosphate buffer, 100 mM pH 7.0 (2/98) and was delivered at 0.9 ml/min. M1 analysis was performed using a MN phenyl column (70 × 4.0 mm). The mobile phase consisted of a mixture of acetonitrile and orthophosphoric acid, 50 mM (15/85) pH 3.0, and was delivered at 0.8 ml/min. For both TMD and M1, fluorescence was measured with emission and excitation wavelengths set at 275 and 308 nm, respectively. In all cases, 50 µl samples were directly injected into the HPLC system. Method performances in terms of reproducibility, repeatability and linearity were assessed before analysis (data not shown). Rhodamine 123 analysis was performed as previously described [24].

In all cases, the samples consisted of aliquots removed from the receiver chambers (drug solution in the aqueous buffer HBSS). No additional treatment was needed. No extraction was required and the samples were directly injected into the HPLC system without need for an internal standard.

### Calculations

TEER was calculated from the following equation [31]:


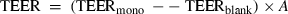


where the TEER_mono_is the cell monolayer and polycarbonate porous membrane resistance, TEER_blank_ the polycarbonate porous membrane resistance and *A* the polycarbonate porous membrane surface area (1.13 cm^2^).

Apical to basolateral (*P*_app_ (A – B)) and basolateral to apical (*P*_app_ (B – A)) apparent permeability coefficients were calculated according to Artursson [32] using the following equation:


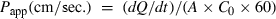


Where *dQ*/*dt* (µg/min) is the permeability rate of the drug, calculated from the regression line of the time points of sampling, *A* is the surface area of the monolayer (cm^3^) and *C*_0_ the initial drug concentration in the donor chamber (µg/l).

Karlsson [33] suggested the involvement of a drug efflux transporter in the investigated Caco-2 cells if the efflux ratio (TR = *P*_app_(B – A)/*P*_app_(A – B)) is >2 and if a decreased secretory transport rate (*P*_app_(B – A)) is observed in the presence of an inhibitor of this transporter. For a compound with an efflux ratio of 1.5–2.0, a positive effect of the inhibitor confirm the implication of the efflux transporter [34].

### Statistics

The Unpaired Student test was used for statistical comparison of the transport rates in each direction, the transport rate in the presence and absence of the **P**-gp inhibitor for a particular direction, the transport efflux ratios in relation to pH conditions and the transport efflux ratio difference in relation to *P*-gp inhibitor addition (Xlstat version 5.0). A P value of <0.05 was considered significant.

## Results

### Integrity of Caco-2 cell monolayers

#### Transepithelial electrical resistance (TEER)

Caco-2 cell monolayers with TEER values between 250 and 350 Ω·cm^2^ were used in the study. Measurements conducted after the experiments displayed similar values and confirmed the integrity of the monolayers during all of the experiments. No tendency towards an effect on TEER was observed under the various experimental conditions (pH, substrates and inhibitors).

#### Paracellular transport of [^3^H]-mannitol

The transport rate of radio-labelled [^3^H]-mannitol was <1% per hr, showing functional tight junctions. As for TEER, no tendency towards an effect on [^3^H]-mannitol permeability was observed under the various experimental conditions (pH, substrates and inhibitors).

### P-glycoprotein activity

#### Transepithelial transport of P-gp probe

**Fig. 2:**
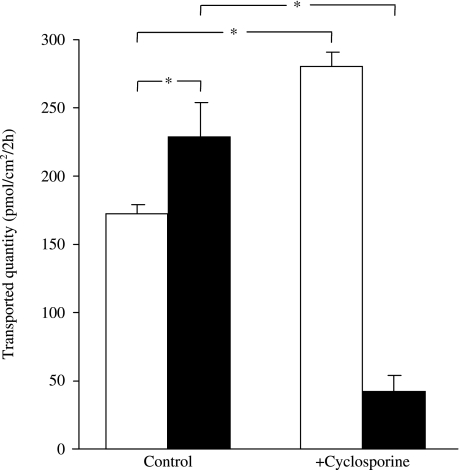
Transepithelial transport of rhodamine 123 across Caco-2 cell monolayers and the effect of cyclosporine A. Rhodamine 123 (5 µM) was added to the apical side (open columns) or the basolateral side (solid columns) of the monolayers in the presence (+cyclosporine) or absence (control) of 10 µM cyclosporine A at pH 6.8/7.4. Data are the mean ± SD of 3 experiments. *P < 0.05.

The *P*-gp probe rhodamine 123 (5 µM) [35] was representative of a good activity of *P*-gp in our Caco-2 cell monolayers. Indeed, our results indicate active transport of rhodamine 123 in the basolateral-to-apical direction (secretion) that exceeds the level of transport in the apical-to-basolateral direction (absorption). Furthermore, in the presence of the *P*-gp inhibitor cyclosporine A (10 µM), we observed a marked increase of the absorptive flux (*P*_app_(A–B): 4.8 ± 0.2 versus 7.8 ± 0.3; P < 0.05) and decrease of the secretory one (*P*_app_(B–A): 6.4 ± 0.7 versus 1.2 ± 0.3; P < 0.05) (fig. 2).

### Transepithelial transport of (+)-TMD, (–)-TMD and M1

#### Transepithelial transport of (+)-TMD and (−)-TMD

**Fig. 3:**
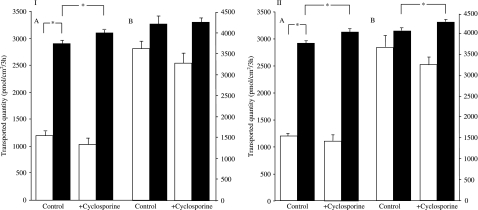
Transepithelial transport of (+)-tramadol and (−)-tramadol across Caco-2 cell monolayers, the effect of cyclosporine A and the influence of pH conditions. (+)-tramadol (I) and (−)-tramadol (II) (10 µM) were added to the apical side (open columns) or the basolateral side (solid columns) of the monolayers in the presence (+cyclosporine) or absence (control) of 10 µM cyclosporine A at pH 6.8/7.4 (A) and pH 7.4/7.4 (B). Data are the mean ± SD of three experiments. *P < 0.05.

Under the pH 6.8/7.4 condition, a significant efflux ratio (TR) of the two enantiomers was observed, with a 1.9 to 2.7-fold higher transepithelial transport from the basolateral to apical side (B–A) than from the apical to basolateral side (A–B). A–B transport was not affected by cyclosporine A, however, although not statistically significant, a trend towards a decrease of the influx (A–B) (up to 13%) was observed. A statistically significant increase of the efflux (B–A) (up to 18%, at all concentrations except 100 µM), and thus of the efflux ratio, was observed in the presence of cyclosporine A (P < 0.05) (table 1, fig. 3).

**Table 1:** Apparent permeability coefficients, *P*_app_ (cm/sec.), of (+)-TMD, (−)-TMD and M1 in the presence and absence of cyclosporine A, at pH 6.8/7.4 and pH 7.4/7.4.

	*P*_app_ (A – B) *P*_app_ (B – A)			*P*_app_ (A – B) *P*_app_ (B – A)		
	(cm/sec.) (10^−6^)			(cm/sec.) (10^−6^)		
Drug concentration ± *P*-gp inhibitor	pH 6.8/7.4		Efflux ratio (TR)	pH 7.4/7.4		Efflux ratio (TR)
**(+)-TMD**						
1 µM	14.1 ± 0.8	31.1 ± 0.8*	2.2 (efflux)	28.1 ± 0.5	31.5 ± 1.3	1.1 (no net flux)‡
+ 10 µM Cyclosporine A	15.6 ± 1.2	34.0 ± 0.7†	2.1	26.2 ± 3.4	34.6 ± 0.8†	1.3
10 µM	11.0 ± 0.7	26.9 ± 0.5*	2.4 (efflux)	33.5 ± 1.4	38.8 ± 1.6	1.1 (no net flux)‡
+ 10 µM Cyclosporine A	9.6 ± 1.1	28.8 ± 0.6†	3.0§	30.2 ± 2.1	39.4 ± 0.8	1.3
50 µM	10.3 ± 0.5	25.6 ± 2.3*	2.4 (efflux)	52.5 ± 1.1	65.3 ± 1.0*	1.2 (no net flux)‡
+ 10 µM Cyclosporine A	9.3 ± 0.6	31.2 ± 0.7†	3.3§	49.9 ± 2.5	69.6 ± 0.7†	1.3§
100 µM	22.6 ± 1.9	60.9 ± 1.3*	2.6 (efflux)	41.7 ± 3.6	66.9 ± 0.4*	1.6 (no net flux)‡
+ 10 µM Cyclosporine A	21.7 ± 1.9	61.7 ± 2.0	2.8	39.1 ± 1.5	68.2 ± 0.9	1.7
**(−)-TMD**						
1 µM	16.7 ± 0.5	32.2 ± 0.5*	1.9 (efflux)	31.0 ± 1.9	30.8 ± 1.8	0.9 (no net flux)‡
+ 10 µM Cyclosporine A	18.7 ± 1.1†	36.0 ± 0.3†	1.9	29.2 ± 1.1	34.2 ± 0.8†	1.1§
10 µM	11.0 ± 0.5	26.9 ± 0.5*	2.4 (efflux)	33.5 ± 2.2	37.2 ± 0.4	1.1 (no net flux)‡
+ 10 µM Cyclosporine A	10.2 ± 1.0	28.9 ± 0.5†	2.8§	29.9 ± 1.5	39.0 ± 0.6†	1.3§
50 µM	10.3 ± 0.5	25.4 ± 2.3*	2.4 (efflux)	52.9 ± 1.0	63.4 ± 3.8	1.1 (no net flux)‡
+ 10 µM Cyclosporine A	9.4 ± 0.6	31.2 ± 0.8†	3.3§	50.9 ± 1.7	68.9 ± 0.4	1.3
100 µM	22.4 ± 1.8	61.3 ± 1.0*	2.7 (efflux)	41.1 ± 2.9	64.3 ± 3.4*	1.5 (no net flux)‡
+ 10 µM Cyclosporine A	21.7 ± 2.0	62.5 ± 2.0	2.8	37.3 ± 1.0	67.7 ± 1.5	1.8
**M1**						
1 µM	8.3 ± 0.3	16.4 ± 0.5*	1.9 (efflux)	18.0 ± 0.9	17.7 ± 0.0	0.9 (no net flux)‡
+ 10 µM Cyclosporine A	7.3 ± 0.2†	21.2 ± 0.6†	2.9	18.2 ± 0.5	19.3 ± 0.5†	1.0§
10 µM	6.5 ± 0.3	12.0 ± 0.8*	1.8 (efflux)	12.9 ± 1.1	14.2 ± 0.8	1.1 (no net flux)‡
+ 10 µM Cyclosporine A	6.8 ± 0.3	15.1 ± 0.5†	2.2§	12.8 ± 0.9	15.0 ± 0.3	1.1
50 µM	5.8 ± 1.0	13.7 ± 0.3*	2.3 (efflux)	15.2 ± 0.4	17.4 ± 0.6	1.1 (no net flux)‡
+ 10 µM Cyclosporine A	5.3 ± 0.5	16.1 ± 0.7†	3.0§	15.4 ± 1.0	20.1 ± 2.4	1.3
100 µM	6.7 ± 0.4	15.2 ± 0.0*	2.2 (efflux)	12.2 ± 0.9	13.7 ± 0.1	1.1 (no net flux)‡
+ 10 µM Cyclosporine A	6.8 ± 0.7	20.6 ± 0.3†	3.0§	11.7 ± 1.1	15.2 ± 0.7†	1.2

The efflux ratio shown here is the net basolateral to apical flux direction (TR = *P*_app_(B–A)/*P*_app_(A–B)).

* P < 0.05. Significant difference in transport direction.

† P < 0.05. Significance of the inhibitor effect for a given transport direction.

‡ P < 0.05. Significant difference in transport efflux ratios in relation to pH conditions.

§ P < 0.05. Significant difference in transport efflux ratios in relation to inhibitor addition for a given pH condition.

Values are the mean ± SD of three experiments.

At physiological pH, the differential transport was nearly abolished (0.9 < TR < 1.2). As for the pH gradient condition, the A–B transport was not significantly affected by cyclosporine A. However, an overall statistically significant increase of the efflux (B–A) (up to 10%), with a slight increase of the efflux ratio, was observed in the presence of cyclosporine A, although this was less pronounced than under the pH gradient condition (P < 0.05) (table 1, fig. 3).

At physiological pH, the A–B transport of (+)-TMD and (−)-TMD was not modulated by GF120918 at the lowest concentration (1 µM), however, a statistically significant increase of the efflux (B–A) (17%) was observed (P < 0.05) (table 2).

**Table 2:** Apparent permeability coefficients, *P*_app_ (cm/sec.), of (+)-TMD, (−)-TMD and M1 in the presence and absence of GF120918 at pH 7.4/7.4.

	*P*_app_ (A–B) *P*_app_ (B–A)		
	(cm/sec.) (10^−6^)		
Drug concentration ± *P*-gp inhibitor	pH 7.4/7.4		Efflux ratio (TR)
**(+)-TMD**			
1 µM	42.6 ± 6.2	52.3 ± 2.9	1.2 (no net flux)
+ 4 µM GF120918	45.8 ± 3.7	60.6 ± 2.1*	1.3
**(−)-TMD**			
1 µM	43.8 ± 5.8	51.1 ± 1.9	1.1 (no net flux)
+ 4 µM GF120918	44.8 ± 4.0	61.3 ± 2.8*	1.3
**M1**			
1 µM	16.9 ± 1.3	16.5 ± 0.7	0.9 (no net flux)
+ 4 µM GF120918	13.5 ± 1.3*	16.2 ± 0.5	1.2

The efflux ratio shown here is the net basolateral to apical flux direction (TR = *P*_app_(B–A)/*P*_app_(A–B)).

* P < 0.05. Significance of the inhibitor effect for a given transport direction.

Values are the mean ± SD of three experiments.

#### Transepithelial transport of M1

**Fig. 4:**
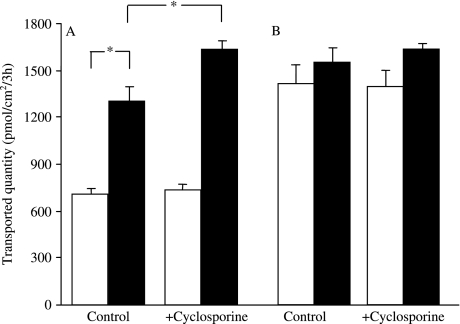
Transepithelial transport of *O*-desmethyl-tramadol across Caco-2 cell monolayers, the effect of cyclosporine A and the influence of pH conditions. *O*-desmethyl-tramadol (10 µM) was added to the apical side (open columns) or the basolateral side (solid columns) of the monolayers in the presence (+cyclosporine) or absence (control) of 10 µM cyclosporine A at pH 6.8/7.4 (A) and pH 7.4/7.4 (B). Data are the mean ± SD of three experiments. *P < 0.05.

Under the pH 6.8/7.4 condition, a significant efflux ratio was observed with a 1.8 to 2.3-fold higher transepithelial transport from the basolateral to apical side (B–A) than from the apical to basolateral side (A–B). The A–B transport was not affected by cyclosporine A, except for a statistically significant decrease observed at 1 µM. A statistically significant increase of the efflux (B–A) (up to 26%) at all concentrations, and thus of the efflux ratio, was observed in the presence of cyclosporine A (P < 0.05) (table 1, fig. 4).

At physiological pH, the differential transport was nearly abolished (0.9 < TR < 1.1). The A–B transport was not affected by cyclosporine A. An overall statistically significant increase of the efflux (B–A) (up to 14%), although less pronounced than at the pH gradient condition, was observed (P < 0.05) (table 1, fig. 4).

At physiological pH, the B–A transport of M1 was not modulated by GF120918 at the lowest concentration (1 µM), however, a statistically significant decrease of the influx (A–B) (20%) was observed (P < 0.05) (table 2).

## Discussion

Our study demonstrates that the two enantiomers of TMD as well as its active opioid metabolite M1 are not *P*-gp substrates. Indeed, a significant secretory transport of the three compounds was observed in the presence of a pH gradient (TR: 1.8–2.7; P < 0.05), suggesting the involvement of an efflux transporter. However, the observed polarised transport was abolished at physiological pH. Furthermore, no effect of the first generation and non-specific *P*-gp inhibitor cyclosporine A and the more potent and specific inhibitor third generation GF120918, in the way of an increase of the absorptive flux (*P*_app_(A–B)) and a decrease of the secretory flux (*P*_app_(B–A)), at both pH conditions was observed. Our *P*-gp positive control rhodamine 123 showed a polarised transport across the Caco-2 monolayers in the way of a net active secretion as well as a 80% decrease of its secretory transport and 38% increase of the absorptive one in the presence of the *P*-gp inhibitor cyclosporine A. Consequently, TMD enantiomers and M1 are not *P*-gp substrates, at respectively 1–100 and 4–400 times the maximal plasma concentrations (Cmax) that produce a clear-cut analgesic effect after a single oral dose of 100 mg TMD racemate [25].

These experimental results are in accordance with the results of pharmacogenetic clinical studies evaluating the role of *P*-gp in TMD kinetics. In fact, although a role of the *P*-gp polymorphism C3435T in the pharmacokinetics of TMD has been suggested in a small clinical trial involving patients with selected CYP2D6 and MDR1 polymorphisms, no statistically significant difference in kinetics was found between the 3435CC, CT and TT groups, independently of CYP2D6 polymorphisms, nor was influence of the G2677T/A polymorphism observed. A significant difference between the pooled CC + CT and TT patients was found only in the CYP2D6 poor metabolizers, and concerned the Cmax parameter only. M1 pharmacokinetics was found to be MDR1 polymorphism-independent [36].

The Caco-2 cell model is one of the most commonly used high throughput and cost-effective *in vitro* models for evaluating permeability and *P*-gp interaction potential of drug candidates. This model is recommended by the Food and Drug Administration (FDA) for the identification of substrates, inhibitors and inducers of *P*-gp through bidirectional transport studies [37,38]. The calibration of the model with appropriate reference probes and a thorough understanding of the rationale underlying the caveats associated with this model allow the results to be put in the proper perspective [24,39]. The Caco-2 cell monolayers are moreover a good model for pH-gradient transport studies for the following reasons: (i) the monolayers remain intact at apical pH values that cover the entire physiological range of the intestinal tract; (ii) they lack a protective mucus layer, which means that the pH at the cell surface rapidly equilibrates and becomes equal to the bulk pH of the applied buffer solution [40].

In our study, (+)TMD, (−)-TMD and M1 permeability in the apical-to-basolateral (A–B) direction (absorptive flux) decreased approximately by 50% when apical pH was reduced from pH 7.4 to 6.8, resulting in a significant efflux ratio of the drugs (TR: *P*_app_(B–A)/*P*_app_(A–B): 1.8–2.7; P < 0.05). TMD and M1 are weak bases. This pH dependency of TMD and M1 absorptive flux is not explained by the passive diffusion of the unionised form according to the pH-partition theory. Indeed, the pKa of TMD is 9.4 and pH values in our study are among 2 and 2.4 units below its pKa. Hence, 100% of TMD is in the ionised form at both pH 6.8 and 7.4. M1 pKa is unknown but it is expected to be close to that of TMD. Therefore, the polarised transport of (+)-TMD, (−)-TMD and M1 observed under a pH gradient is probably related to the contribution of specific efflux transport system(s). The overall effect of the *P*-gp inhibitors used in our study on the transepithelial transport of the two enantiomers of TMD and M1 was a statistically significant increase of the secretory flux (up to 18% and 26%, respectively), with an increase of the efflux ratio (TR), at both pH conditions. A higher increase of the secretory flux of TMD enantiomers was observed with GF120918 at physiological pH as compared with cyclosporine A (17% versus 10%). Moreover, a significant decrease of the absorptive flux of M1 (approximately 20%) was observed in the presence of GF120918 while no effect was observed with cyclosporine A. This apparent paradoxical effect of *P*-gp inhibitors suggests the involvement of uptake drug transporter(s) in the transmembrane transport of TMD enantiomers and M1.

The gastrointestinal tract and the kidney provide a unique environment of cellular transport due to the presence of proton-based efflux pumps such as the Na^+^–H^+^ and the organic cation/H^+^exchangers [26,27]. The Na^+^–H^+^ antiporter has been characterized in Caco-2 cells [41] and studies using the Caco-2 cell model showed an active secretory transport of weak bases such as celiprolol, loperamide, zolmitriptan, diphenydramine and amphetamine derivatives by proton-based efflux pumps [33,42–45]. H^+^antiport systems might therefore be involved in the limitation of the gastrointestinal absorption of TMD as well as renal excretion of TMD and M1. The use of specific inhibitors such as amiloride, or cimetidine could help confirming this hypothesis [33,42–45]. Apart from efflux drug transporters, Caco-2 cells express several uptake transporters. The most relevant are the organic anion transporting polypeptide OATP2B1/SLCO2B1, the peptide transporter PEPT1/SLC15A1, the organic cation/carnitine OCNT2/SLC22A5 as well as the monocarboxylic acid transporters MCT1/SLC16A1 and MCT5/SLC16A5 and the human peptide transporter HPT1/CDH17 [46,47]. PEPT1 is inhibited by cyclosporine A [48]. Moreover, it has been shown to transport endogenous opioid peptides and consequently might be relevant in the oral bioavailability of synthetic opioids [49].

TMD and M1 cross easily the blood–brain barrier [25]. They appear to cross the cellular membrane efficiently and to accumulate in tissues, and these pharmacokinetic properties are in concordance with our study results. Indeed, a comparative study of Caco-2 monolayer permeability and human drug absorption suggests that compounds with *P*_app_ > 10 × 10^−6^ cm/sec. can be classified as well-absorbed compounds (70–100%) [50]. Since TMD (pKa: 9.4) and M1 are almost 100% protonated at both pH conditions tested, a passive diffusion mechanism may not fully explain TMD and M1 high tissue distribution. The involvement of uptake transporters seems therefore relevant in this case. More specific *in vitro* models are required to support our observations.

In conclusion, (+)-TMD, (−)-TMD and the major analgesic metabolite *O*-desmethyl-TMD are not *P*-gp substrates. However, proton-based efflux pumps may be involved in limiting the gastrointestinal absorption of TMD enantiomers and as well as enhancing TMD enantiomers and M1 renal excretion. Moreover, our investigations suggest the possible involvement of uptake carriers in their transepithelial transport across biological membranes. The hypothesis advanced, which might be of importance in the prediction of clinically significant drug interactions, needs to be investigated.

## References

[1] Schenck EG, Arend I (1978). [The effect of tramadol in an open clinical trial (author’s transl)]. Arzneimittelforschung.

[2] Lehmann KA (1994). Tramadol for the management of acute pain. Drugs.

[3] Savoia G, Loreto M, Scibelli G (2000). [Systemic review of trials on the use of tramadol in the treatment of acute and chronic pain]. Minerva Anestesiol.

[4] Scott LJ, Perry CM (2000). Tramadol: a review of its use in perioperative pain. Drugs.

[5] Lintz W, Erlacin S, Frankus E, Uragg H (1981). [Biotransformation of tramadol in man and animal (author's transl)]. Arzneimittelforschung.

[6] Paar WD, Frankus P, Dengler HJ (1992). The metabolism of tramadol by human liver microsomes. Clin Investig.

[7] Raffa RB, Friderichs E, Reimann W, Shank RP, Codd EE, Vaught JL (1993). Complementary and synergistic antinociceptive interaction between the enantiomers of tramadol. J Pharmacol Exp Ther.

[8] Gillen C, Haurand M, Kobelt DJ, Wnendt S (2000). Affinity, potency and efficacy of tramadol and its metabolites at the cloned human mu-opioid receptor. Naunyn Schmiedebergs Arch Pharmacol.

[9] Radbruch L, Grond S, Lehmann KA (1996). A risk-benefit assessment of tramadol in the management of pain. Drug Saf.

[10] Poulsen L, Arendt-Nielsen L, Brosen K, Sindrup SH (1996). The hypoalgesic effect of tramadol in relation to CYP2D6. Clin Pharmacol Ther.

[11] Stamer UM, Lehnen K, Hothker F, Bayerer B, Wolf S, Hoeft A (2003). Impact of CYP2D6 genotype on postoperative tramadol analgesia. Pain.

[12] Wang G, Zhang H, He F, Fang X (2006). Effect of the CYP2D6*10 C188T polymorphism on postoperative tramadol analgesia in a Chinese population. Eur J Clin Pharmacol.

[13] Kirchheiner J, Keulen JT, Bauer S, Roots I, Brockmöller J (2008). Effects of the CYP2D6 gene duplication on the pharmacokinetics and pharmacodynamics of tramadol. J Clin Psychopharmacol.

[14] Campanero MA, Calahorra B, Valle M, Troconiz IF, Honorato J (1999). Enantiomeric separation of tramadol and its active metabolite in human plasma by chiral high-performance liquid chromatography: application to pharmacokinetic studies. Chirality.

[15] Liu HC, Liu TJ, Yang YY, Hou YN (2001). Pharmacokinetics of enantiomers of trans-tramadol and its active metabolite, trans-*O*-demethyltramadol, in human subjects. Acta Pharmacol Sin.

[16] DuBuske LM (2005). The role of *P*-glycoprotein and organic anion-transporting polypeptides in drug interactions. Drug Safety.

[17] Siccardi D, Kandalaft LE, Gumbleton M, McGuigan C (2003). Stereoselective and concentration-dependent polarized epithelial permeability of a series of phosphoramidate triester prodrugs of d4T: an *in vitro* study in Caco-2 and Madin-Darby canine kidney cell monolayers. J Pharmacol Exp Ther.

[18] Comets E, Gautrand C, Fernandez C, Auchere D, Singlas E, Barraud dL (2004). Cerebral uptake of mefloquine enantiomers with and without the *P*-gp inhibitor elacridar (GF1210918) in mice. Br J Pharmacol.

[19] Miura M, Uno T, Tateishi T, Suzuki T (2007). Pharmacokinetics of fexofenadine enantiomers in healthy subjects. Chirality.

[20] Wang JS, Ruan Y, Taylor RM, Donovan JL, Markowitz JS, DeVane CL (2004). Brain penetration of methadone (R)- and (S)-enantiomers is greatly increased by *P*-glycoprotein deficiency in the blood–brain barrier of Abcb1a gene knockout mice. Psychopharmacology (Berl).

[21] Crowe A (2002). The influence of *P*-glycoprotein on morphine transport in Caco-2 cells. Comparison with paclitaxel. Eur J Pharmacol.

[22] Wandel C, Kim R, Wood M, Wood A (2002). Interaction of morphine, fentanyl, sufentanil, alfentanil, and loperamide with the efflux drug transporter *P*-glycoprotein. Anesthesiology.

[23] Hassan HE, Myers AL, Lee IJ, Coop A, Eddington ND (2007). Oxycodone induces overexpression of *P*-glycoprotein (ABCB1) and affects paclitaxel's tissue distribution in Sprague Dawley rats. J Pharm Sci.

[24] Kanaan M, Daali Y, Dayer P, Desmeules J (2008). Lack of interaction of the NMDA receptor antagonists dextromethorphan and dextrorphan with *P*-glycoprotein. Curr Drug Metab.

[25] Grond S, Sablotzki A (2004). Clinical pharmacology of tramadol. Clin Pharmacokinet.

[26] Tsuji A, Tamai I (1996). Carrier-mediated intestinal transport of drugs. Pharm Res.

[27] Inui KI, Masuda S, Saito H (2000). Cellular and molecular aspects of drug transport in the kidney. Kidney Int.

[28] Löscher W, Potschka H (2005). Blood-brain barrier active efflux transporters: ATP-binding cassette gene family. NeuroRx.

[29] Taipalensuu J, Törnblom H, Lindberg G, Einarsson C, Sjöqvist F, Melhus H (2001). Correlation of gene expression of ten drug efflux proteins of the ATP-binding cassette transporter family in normal human jejunum and in human intestinal epithelial Caco-2 cell monolayers. J Pharmacol Exp Ther.

[30] Ardakani YH, Mehvar R, Foroumadi A, Rouini MR (2008). Enantioselective determination of tramadol and its main phase I metabolites in human plasma by high-performance liquid chromatography. J Chromatogr B Analyt Technol Biomed Life Sci.

[31] Amidon GL, Lennernas H, Shah VP, Crison JR (1995). A theoretical basis for a biopharmaceutic drug classification: the correlation of *in vitro* drug product dissolution and *in vivo* bioavailability. Pharm Res.

[32] Artursson P (1990). Epithelial transport of drugs in cell culture. I: A model for studying the passive diffusion of drugs over intestinal absorptive (Caco-2) cells. J Pharm Sci.

[33] Karlsson J, Kuo SM, Ziemniak J, Artursson P (1993). Transport of celiprolol across human intestinal epithelial (Caco-2) cells: mediation of secretion by multiple transporters including P-glycoprotein. Br J Pharmacol.

[34] Polli JW, Wring SA, Humphreys JE, Huang L, Morgan JB, Webster LO (2001). Rational use of *in vitro P*-glycoprotein assays in drug discovery. J Pharmacol Exp Ther.

[35] Takano M, Hasegawa R, Fukuda T, Yumoto R, Nagai J, Murakami T (1998). Interaction with *P*-glycoprotein and transport of erythromycin, midazolam and ketoconazole in Caco-2 cells. Eur J Pharmacol.

[36] Lötsch J, Skarke C, Liefhold J, Geisslinger G (2004). Genetic predictors of the clinical response to opioid analgesics: clinical utility and future perspectives. Clin Pharmacokinet.

[37] US Food and Drug Administration Draft guidance for industry: Drug interaction studies – study design, data analysis, and implications for dosing and labelling. http://www.fda.gov/cder/guidance.6695dft.pdf.

[38] Zhang L, Strong JM, Qiu W, Lesko LJ, Huang SM (2006). Scientific perspectives on drug transporters and their role in drug interactions. Mol Pharm.

[39] Sun H, Chow EC, Liu S, Du Y, Pang KS (2008). The Caco-2 cell monolayer: usefulness and limitations. Expert Opin Drug Metab Toxicol.

[40] Neuhoff S, Ungell AL, Zamora I, Artursson P (2003). pH-dependent bidirectional transport of weakly basic drugs across Caco-2 monolayers: implications for drug–drug interactions. Pharm Res.

[41] Watson AJ, Levine S, Donowitz M, Montrose MH (1991). Kinetics and regulation of a polarized Na(+)–H+ exchanger from Caco-2 cells, a human intestinal cell line. Am J Physiol.

[42] Crowe A, Wong P (2004). pH dependent uptake of loperamide across the gastrointestinal tract: an *in vitro* study. Drug Dev Ind Pharm.

[43] Yu L, Zeng S (2007). Transport characteristics of zolmitriptan in a human intestinal epithelial cell line Caco-2. J Pharm Pharmacol.

[44] Mizuuchi H, Katsura T, Hashimoto Y, Inui K (2000). Transepithelial transport of diphenhydramine across monolayers of the human intestinal epithelial cell line Caco-2. Pharm Res.

[45] Crowe A, Diep S (2008). pH dependent efflux of methamphetamine derivatives and their reversal through human Caco-2 cell monolayers. Eur J Pharmacol.

[46] Seithel A, Karlsson J, Hilgendorf C, Bjorquist A, Ungell AL (2006). Variability in mRNA expression of ABC- and SLC-transporters in human intestinal cells: comparison between human segments and Caco-2 cells. Eur J Pharm Sci.

[47] Hilgendorf C, Ahlin G, Seithel A, Artursson P, Ungell AL, Karlsson J (2007). Expression of thirty-six drug transporter genes in human intestine, liver, kidney, and organotypic cell lines. Drug Metab Dispos.

[48] Motohashi H, Katsura T, Saito H, Inui K (2001). Effects of tacrolimus and cyclosporin A on peptide transporter PEPT1 in Caco-2 cells. Pharm Res.

[49] Ganapathy V, Miyauchi S (2005). Transport systems for opioid peptides in mammalian tissues. AAPS J.

[50] Yee S (1997). *In vitro* permeability across Caco-2 cells (colonic) can predict *in vivo* (small intestinal) absorption in man: fact or myth. Pharm Res.

